# A Sensor-Based TinyML Acoustic Monitoring System for Edge-Side Animal Sound Recognition on Resource-Constrained Microcontrollers

**DOI:** 10.3390/s26133972

**Published:** 2026-06-23

**Authors:** Zhiqing Wang, Guicai Yu

**Affiliations:** School of Intelligent Science and Engineering, Qinghai Minzu University, Xining 810007, China; 20253601073@qhmu.edu.cn

**Keywords:** animal acoustic monitoring, TinyML, edge artificial intelligence, microcontroller, MFCC, feature consistency, sensor-based recognition

## Abstract

**Highlights:**

**What are the main findings?**

- A microcontroller-based acoustic sensing system was implemented for TinyML animal sound recognition using an onboard PDM microphone.
- A PC-to-microcontroller feature-consistency processing chain was constructed for MFCC extraction and deployment-side standardization.

**What are the implications of the main findings?**

- The proposed system provides a feasible edge-side solution for low-maintenance acoustic monitoring under resource-constrained deployment conditions.
- The results show that feature-consistent preprocessing and source-isolated evaluation are important for reliable microcontroller-level bioacoustic recognition.

**Abstract:**

Edge-side acoustic monitoring enables animal sound recognition in remote environments, but microcontroller deployment remains constrained by feature extraction, numerical consistency, memory, latency, and energy consumption. This study presents a sensor-based tiny machine learning (TinyML) acoustic monitoring system on an Arduino Nano 33 BLE Sense Rev2 platform, integrating onboard pulse-density modulation (PDM) microphone acquisition, Mel-frequency cepstral coefficient (MFCC) feature extraction, deployment-side standardization, 8-bit integer (INT8) neural-network inference, and edge-side decision output. To reduce training-to-deployment feature drift, consistent frame parameters, mirrored C++ feature operators, and exported standardization parameters are used to align personal-computer-side and microcontroller-side feature representations. A source-isolated seven-class protocol was constructed for six target animal classes and one compound background-noise class. In the single-run baseline comparison, the proposed multilayer perceptron achieved 98.28% test accuracy and 97.21% test macro-F1, while the ten-seed stability analysis yielded 98.64% ± 0.26% test accuracy and 97.87% ± 0.38% test macro-F1. The deployed INT8 model occupied approximately 26.9 KB, with a post-window latency of about 303 ms. System-level input power was 0.783–0.825 W, corresponding to an estimated autonomy of 7.63–8.03 h under the reference battery setting.

## 1. Introduction

The Qinghai–Tibet Plateau is a critical ecological security barrier in Asia and provides habitats for several representative wildlife species, including white-lipped deer, Tibetan antelope, Tibetan wild ass, wild yak, leopard, and snow leopard [1]. Long-term monitoring of these species is important for biodiversity conservation and ecological assessment in high-altitude environments. However, continuous wildlife monitoring in this region is constrained by complex terrain, low temperature, hypoxia, long maintenance intervals, and the high cost of manual patrols. Although passive acoustic monitoring can provide useful soundscape evidence for ecological observation, conventional acoustic-recording workflows usually depend on periodic data retrieval, offline screening, and centralized analysis [2,3,4,5]. These limitations motivate the development of low-power, low-maintenance, and edge-side acoustic monitoring systems that can perform local recognition on distributed acoustic sensing nodes.

Recent advances in microelectromechanical systems (MEMS) microphones, low-power microcontrollers, and embedded machine learning have made tiny machine learning (TinyML) a feasible approach for field-deployed acoustic sensing [6,7,8]. Compared with the conventional workflow of continuous acquisition, storage, backhaul, and offline analysis, a microcontroller-based acoustic recognition node can perform sound acquisition, feature extraction, and model inference locally, thereby reducing raw-audio transmission and improving response efficiency [6,9,10]. Nevertheless, deploying animal acoustic recognition on resource-constrained microcontrollers remains challenging. Static random-access memory (SRAM), flash storage, computational throughput, and runtime latency are highly limited on such platforms, requiring a careful balance among acoustic feature representation, model size, inference speed, and system stability [6,8,9,10,11].

Another key challenge is the mismatch between the personal computer (PC)-side training pipeline and the microcontroller-side deployment pipeline. During model development, acoustic features such as Mel-frequency cepstral coefficients (MFCCs) are often extracted using high-level audio-processing libraries [12,13]. In contrast, deployment on microcontrollers requires the same feature-extraction procedure to be reimplemented using C/C++ or low-level digital signal-processing operators. Differences in fast Fourier transform scaling, windowing, Mel filter-bank construction, log-energy computation, discrete cosine transform, floating-point precision, and standardization-parameter application may lead to cross-platform feature drift [13,14]. Such drift can shift the model input distribution during deployment and reduce the reliability of training-to-device transfer.

Complex field soundscapes further increase the difficulty of microcontroller-level animal acoustic recognition. Remote high-altitude environments contain non-stationary background components such as strong wind, rainfall, thunder, vegetation friction, distant human speech, and mechanical noise. These sounds may mask target vocalizations, distort MFCC feature morphology, and introduce abnormal high-amplitude feature responses [4,5,15,16,17]. Therefore, edge-side animal acoustic monitoring requires not only a lightweight classification model, but also a sensing-chain-aware design that considers hardware acquisition characteristics, feature consistency, background-noise coverage, and source-isolated evaluation.

To address these issues, this study develops a sensor-based TinyML acoustic monitoring system for edge-side animal sound recognition on resource-constrained microcontrollers. The system is deployed on the Arduino Nano 33 BLE Sense Rev2 platform (Arduino S.r.l., Monza, Italy) and integrates onboard pulse-density modulation (PDM) microphone acquisition, MFCC feature extraction, feature standardization, 8-bit integer (INT8)-quantized neural-network inference, and edge-side recognition output. The main objective is to validate whether a compact sensor-based recognition pipeline can support animal acoustic monitoring under microcontroller-level memory and latency constraints while reducing training-to-deployment feature inconsistency.

The main contributions of this study are summarized as follows. First, a complete sensor-based TinyML acoustic monitoring system is designed for microcontroller-level animal sound recognition, integrating onboard PDM microphone acquisition, MFCC feature extraction, feature standardization, INT8 neural-network inference, and edge-side decision output on an Arduino Nano 33 BLE Sense Rev2 platform. Second, a cross-platform feature-consistency pipeline is constructed by aligning MFCC extraction parameters, mirrored C++ feature operators, and exported standardization parameters between the training-side and deployment-side processing chains, thereby reducing numerical feature drift before inference. Third, a source-isolated seven-class evaluation protocol is established using six target animal classes and a compound background-noise class, and the system is assessed through Quick Clean threshold analysis, baseline model comparison, multi-seed stability evaluation, and lightweight convolutional neural network (CNN) comparison. Fourth, the edge-side deployment behavior is characterized through microcontroller resource usage, post-window latency, root-mean-square (RMS) trigger-threshold sensitivity, system-level power consumption, and estimated battery autonomy. Finally, the behavior of the classifier under unknown non-target acoustic inputs is analyzed to clarify the boundary between compound background-noise classification and open-set acoustic rejection.

## 2. Related Work

### 2.1. Sensor-Based Bioacoustic Monitoring

Passive acoustic monitoring has become an important technique for biodiversity assessment and wildlife monitoring, particularly in environments where continuous manual observation is difficult, costly, or ecologically intrusive. Compared with traditional field surveys, acoustic sensing systems can collect long-duration soundscape records and provide complementary information for monitoring vocalizing or acoustically active species [2,3]. This characteristic is especially relevant to high-altitude and remote ecological regions, where complex terrain, low temperature, hypoxia, and long maintenance intervals make continuous human patrols difficult. In such contexts, sensor-based acoustic monitoring can support long-term ecological observation by reducing dependence on continuous human presence and enabling the accumulation of environmental acoustic evidence [1,2,3].

Related acoustic-monitoring systems have also been developed in precision livestock farming. Ntalampiras et al. presented an integrated acoustic monitoring system for goat farms, showing how animal sound monitoring can be organized as a farm-level sensing and information system [18]. Their work further illustrates the importance of integrating acoustic sensing, environmental variability, and practical deployment requirements in real-world animal-monitoring systems.

Recent advances in computational bioacoustics have further improved the feasibility of automatic animal sound detection and classification. Deep-learning-based systems such as ANIMAL-SPOT and BirdNET have demonstrated the potential of neural networks for animal-independent signal detection, avian diversity monitoring, and large-scale bioacoustic analysis [4,15]. Other studies have investigated animal acoustic classification using raw waveforms or learned acoustic representations, showing that data-driven models can capture discriminative patterns from complex animal vocalizations [16]. In parallel, reviews of computational bioacoustics have emphasized that deep learning has become an important methodological direction for automatic species identification, acoustic event detection, and ecological soundscape analysis [5].

Despite these advances, practical bioacoustic monitoring remains constrained by complex field soundscapes. Real-world acoustic scenes often contain overlapping vocalizations, strong environmental noise, limited annotated samples, and diverse non-target sounds, all of which can degrade recognition reliability [4,5,15,16]. Moreover, soundscape-based biodiversity monitoring may have limited cross-scene generalizability when acoustic conditions, recording contexts, or ecological environments vary substantially [17]. These limitations suggest that animal acoustic recognition systems should not only optimize classification accuracy under a fixed dataset, but also consider background-noise coverage, cross-scene robustness, and deployment-oriented evaluation protocols. For edge-side acoustic monitoring, this requirement becomes more demanding because the sensing node must perform useful local inference under constrained computation, memory, and energy budgets.

### 2.2. TinyML Audio Recognition on Resource-Constrained Microcontrollers

Tiny machine learning provides a technical pathway for deploying machine-learning inference directly on low-power microcontrollers. TensorFlow Lite Micro and related embedded machine-learning frameworks enable neural-network inference without relying on high-performance processors or continuous cloud connectivity [6,9]. Systematic reviews of TinyML indicate that ultra-low-power learning systems are increasingly important for large-scale Internet of Things sensing, environmental monitoring, and distributed intelligent perception [7,8]. Benchmarking efforts such as MLPerf Tiny also show that embedded machine-learning systems should be evaluated not only in terms of accuracy, but also in terms of latency, memory footprint, and deployment feasibility [10].

Audio recognition on microcontrollers is particularly challenging because acoustic sensing requires continuous sampling, short-time signal processing, feature buffering, and model inference. Unlike high-performance edge platforms, microcontroller systems are usually constrained by limited static random-access memory, flash storage, and computational throughput [6,7,8,9,10,11]. These constraints make it necessary to use compact acoustic representations and lightweight models. Mel-frequency cepstral coefficients (MFCCs) remain widely used in speech and acoustic signal processing because they provide a compact representation of spectral-envelope information while keeping the feature dimensionality moderate [12,19]. For resource-constrained acoustic recognition, MFCC-based features can reduce model input size, feature-buffer overhead, and inference cost compared with raw-waveform or high-resolution spectrogram inputs.

Recent studies have begun to explore microcontroller-level or ultra-low-power acoustic monitoring systems. For example, TinyBird-ML demonstrates an ultra-low-power smart sensor node for bird vocalization analysis and syllable classification, indicating the potential of embedded acoustic intelligence for field monitoring applications [20]. However, many TinyML audio studies still focus on benchmark tasks, keyword spotting, or relatively controlled audio classification scenarios. For animal acoustic monitoring in complex field environments, additional issues arise, including sparse target events, non-stationary background noise, hardware-dependent acoustic acquisition, and the need to validate whether the trained model can be reliably transferred to the deployed sensing device. Therefore, microcontroller-based animal acoustic recognition should be considered as a complete sensing and deployment problem, rather than only as a lightweight model-compression problem.

### 2.3. Cross-Platform Acoustic Feature Consistency and Robust Evaluation

A key challenge in embedded acoustic recognition is the potential inconsistency between the PC-side training pipeline and the microcontroller-side deployment pipeline. During model development, acoustic features are often extracted using high-level software libraries, such as librosa, whereas deployment requires the corresponding feature-extraction procedure to be implemented using C/C++ or low-level digital signal-processing operators on the target microcontroller [13]. Even when the same nominal MFCC configuration is used, implementation differences in fast Fourier transform scaling, windowing, Mel filter-bank construction, log-energy computation, discrete cosine transform, floating-point precision, and standardization-parameter application may lead to numerical or statistical discrepancies in the generated feature vectors [12,13,14,21,22]. Such cross-platform feature drift can shift the input distribution observed by the deployed model and may reduce the reliability of training-to-deployment transfer.

Acquisition-domain mismatch is another important source of performance degradation in acoustic sensing systems. Prior work on acoustic scene classification has shown that mismatched recording devices can affect recognition performance because different microphones and acquisition front ends may introduce different spectral responses under the same sound event [14]. This issue is particularly relevant for microcontroller-based systems, where the onboard microphone, sampling path, quantization characteristics, and embedded front-end implementation may differ from the devices or software pipelines used during dataset construction. Consequently, deployment-oriented acoustic monitoring systems should consider hardware-aware data construction and feature-extraction alignment, so that the training data and the online sensor input share more consistent acquisition and feature-distribution characteristics.

Robust evaluation is also essential for animal acoustic recognition under complex soundscapes. Conventional random splitting may introduce same-source leakage when samples derived from the same recording session, background event, or contiguous slicing sequence appear in both the training and test sets. Such leakage can produce overly optimistic evaluation results and obscure the model’s true generalization capability [23]. For datasets with temporal, spatial, hierarchical, or source-dependent structure, evaluation protocols should account for the underlying dependency structure instead of relying only on random assignment [24]. In bioacoustic monitoring, this issue is especially important because background scenes, recording sessions, device conditions, and environmental contexts may leave source-specific acoustic fingerprints in the data.

Data augmentation and background expansion can improve model training when annotated animal sounds are limited or field conditions are complex [25,26,27]. However, augmentation alone cannot fully replace natural variation across recording devices, distances, seasons, and environmental conditions. Moreover, complex background sounds such as wind, rainfall, thunder, vegetation friction, human speech, and mechanical noise may introduce high-energy interference and feature-domain distortion, thereby reducing separability between target animal sounds and background acoustic events [5,17,28]. Therefore, robust animal acoustic recognition requires not only data expansion, but also careful background modeling, source-aware partitioning, and feature-domain analysis.

Existing studies have provided important foundations for passive acoustic monitoring, deep-learning-based bioacoustic classification, TinyML deployment, and acoustic feature extraction. However, comparatively less attention has been paid to the complete deployment chain in which onboard acoustic sensing, hardware-aware data construction, PC-to-microcontroller feature consistency, source-isolated evaluation, complex-background robustness, and resource-constrained inference are considered together. This gap is particularly relevant for microcontroller-level animal acoustic monitoring, where the sensing front end, feature pipeline, dataset partitioning strategy, and runtime resource budget jointly determine deployment reliability. To address this issue, the present study develops a sensor-based TinyML acoustic monitoring system for resource-constrained microcontrollers, with emphasis on deployment-aligned MFCC extraction, hardware-aware background construction, source-isolated evaluation, and edge-side resource feasibility.

## 3. Materials and Methods

### 3.1. System Architecture and Sensor-Based Edge Deployment

**Figure 1:**
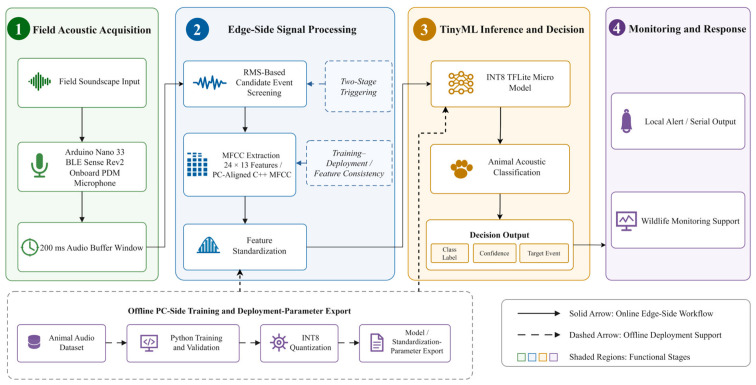
Overall architecture of the proposed sensor-based TinyML acoustic monitoring system for edge-side animal sound recognition.

The overall architecture of the proposed sensor-based TinyML acoustic monitoring system is shown in Figure 1. The system consists of four online functional stages: field acoustic acquisition, edge-side signal processing, TinyML inference, and decision-making, and monitoring and response. In addition, an offline PC-side workflow is used for acoustic feature generation, lightweight model training, INT8 quantization, and deployment-parameter export. The offline workflow provides the quantized model and standardization parameters required for microcontroller-side deployment.

In the online workflow, field acoustic signals are first captured by the onboard pulse-density modulation (PDM) microphone of the Arduino Nano 33 BLE Sense Rev2. The incoming audio stream is organized into fixed-length windows and passed to the edge-side signal-processing module. The signal-processing module performs lightweight acoustic event screening, Mel-frequency cepstral coefficient (MFCC) feature extraction, and feature standardization. The standardized feature vector is then used as the input to an INT8-quantized TinyML model for animal acoustic classification. The output layer provides the predicted class label, confidence information, and target-event decision for local alerting or subsequent monitoring support.

The offline workflow supports the training-to-deployment process. Raw animal sounds and background-noise samples are processed on the PC side using an MFCC procedure configured consistently with the microcontroller-side implementation. The extracted features are used for feature-domain filtering, standardization-parameter estimation, lightweight neural-network training, and post-training INT8 quantization. The trained quantized model and the estimated mean and standard deviation arrays are then exported for deployment on the microcontroller. This workflow is designed to reduce inconsistency between the training-side feature distribution and the deployment-side feature distribution.

Compared with high-performance edge platforms, such as Raspberry Pi or Jetson devices, microcontroller platforms provide much more limited memory, storage, and computational resources. Therefore, the proposed system is designed around the joint constraints of acoustic feature compactness, real-time responsiveness, memory footprint, and on-device deployability. The system does not aim to maximize model complexity. Instead, it emphasizes a complete sensor-side processing loop that can be executed on a resource-constrained microcontroller, including audio acquisition, front-end feature extraction, model inference, and local result output.

### 3.2. Hardware Platform and Real-Time Audio Stream Processing

The target deployment platform is the Arduino Nano 33 BLE Sense Rev2 (Arduino S.r.l., Monza, Italy), which serves as the microcontroller unit (MCU) platform in this study. This board integrates a Nordic nRF52840 system-on-chip (Nordic Semiconductor ASA, Trondheim, Norway) with an Arm Cortex-M4F core and floating-point unit, and an onboard PDM digital microphone, which makes it suitable for lightweight digital signal processing and TinyML inference under embedded resource constraints. In this study, the onboard microphone is used as the primary acoustic sensing front end, so that the deployed system can acquire and process sound signals using the same hardware chain intended for long-term edge monitoring. The deployment firmware was developed using Arduino IDE 1.8.19 with the Arduino mbed_nano core 4.4.1, PDM library 1.0, and TensorFlow Lite Micro 2.4.0-ALPHA.

The acoustic input is sampled at 16 kHz and organized into analysis windows of 200 ms. Each window contains 3200 audio samples. This window length is selected to preserve short-duration animal acoustic patterns while controlling buffering cost and post-window processing latency. After window formation, the system extracts a compact MFCC feature representation and feeds it into the quantized neural-network classifier. The feature dimensionality is fixed at 312, corresponding to 24 analysis frames and 13 MFCC coefficients per frame.

Field animal acoustic events are typically sparse in continuous environmental audio. To avoid unnecessary execution of the full feature-extraction and inference pipeline on silent or low-energy segments, the system adopts a two-stage asynchronous triggering mechanism. In the first stage, lightweight amplitude or root-mean-square energy screening is used to determine whether a candidate acoustic event is present. When the input energy remains below the triggering threshold, the system maintains basic sampling and low-cost signal monitoring. In the second stage, when the triggering condition is satisfied, the system activates MFCC extraction, feature standardization, and TensorFlow Lite Micro inference. This design reduces redundant computation on low-information audio segments and provides a basis for future low-power optimization.

Because audio sampling, feature extraction, and model inference share limited resources on a single-core microcontroller, the system uses a double-buffer-based quasi-non-blocking stream-processing strategy. Two fixed-length audio buffers are organized in a ping-pong structure. While one buffer receives incoming samples, the other buffer can be processed by the main loop after it has been marked as a completed audio window. Once the active write buffer is filled, the system switches to the other buffer for continued sampling. The main loop performs MFCC extraction and neural-network inference only on completed buffers. This strategy temporally decouples audio sampling from downstream computation and reduces the risk that incoming acoustic segments are truncated during feature extraction or model inference.

After a completed window is passed to the feature-extraction module, zero-padding protection is applied when the available input length is shorter than the required analysis length. This mechanism prevents out-of-bounds memory access during frame-level MFCC computation. The raw 312-dimensional MFCC vector is then standardized using the mean and standard deviation parameters exported from the training side and is subsequently used as the model input. By fixing the audio-window length, frame configuration, feature dimensionality, and model input size, the system ensures that the major runtime buffers and tensor shapes can be predetermined before deployment.

### 3.3. MFCC Feature Extraction and Compact Feature Representation

To obtain a compact acoustic representation suitable for microcontroller deployment, this study uses Mel-frequency cepstral coefficient (MFCC) features as the model input representation. MFCCs provide a low-dimensional representation of spectral-envelope characteristics and have been widely used in speech and acoustic signal processing [12,19]. Compared with raw waveform inputs or high-resolution spectrograms, MFCC-based features reduce the model input dimensionality, feature-buffer size, and inference cost, which is important for resource-constrained microcontroller platforms.

For each 200 ms audio window sampled at 16 kHz, the system obtains 3200 audio samples. The input window is divided into short time-frames using a 256-sample frame length and a 128-sample frame hop, corresponding to 50% overlap between adjacent frames. This configuration produces 24 complete analysis frames for each audio window. Each frame is weighted by a Hamming window to reduce spectral leakage, followed by fast Fourier transform (FFT) computation, power-spectrum calculation, Mel filter-bank mapping, natural-logarithm compression, and type-II discrete cosine transform (DCT-II). The number of Mel filters is set to 26, and 13 MFCC coefficients are retained for each frame. Therefore, the final feature representation is organized as a 24 × 13 feature matrix, corresponding to a 312-dimensional feature vector.

The feature dimensionality is defined as

(1) $$D=N_{f}\times N_{c}=24\times 13=312$$

where D denotes the total feature dimensionality, $N_{f}$ denotes the number of analysis frames, and $N_{c}$ denotes the number of MFCC coefficients retained for each frame. The same MFCC parameter configuration is used in both the PC-side training pipeline and the microcontroller-side deployment pipeline, providing the feature-level basis for subsequent consistency alignment.

Table 1 summarizes the MFCC feature-extraction parameters used in this study.

Before model training, the extracted MFCC feature vectors are processed using a feature-domain anomalous-sample filtering rule. This procedure, denoted Quick Clean, is applied only during training and evaluation data preparation and is distinct from the online amplitude-based triggering mechanism used on the microcontroller. Its purpose is to remove feature vectors containing invalid numerical entries or abnormally large feature amplitudes that may distort the training distribution.

For each MFCC feature vector $f=[f_{1},f_{2},\ldots ,f_{D}]$, Quick Clean retains a sample only when the vector contains no not-a-number (NaN) entries and its maximum absolute feature magnitude is below a predefined threshold τ. The feature-domain amplitude constraint is expressed as

(2) $$max_{1\leq i\leq D}\left|f_{i}\right|<\tau$$

In the final experimental setting, τ is set to 200 after threshold calibration. This setting is used to suppress abnormal high-magnitude feature samples while avoiding excessive removal of valid animal acoustic samples under complex background conditions. Since Quick Clean operates on extracted MFCC features rather than on raw waveforms, it does not modify the waveform morphology of any sample; instead, it determines whether a feature vector enters the subsequent training and evaluation pipeline.

### 3.4. PC-to-Microcontroller Feature-Consistency Alignment

A central objective of the proposed system is to reduce feature drift between the PC-side training pipeline and the microcontroller-side deployment pipeline. During training, acoustic features can be computed using a reference implementation on a personal computer, whereas deployment requires the same feature-extraction logic to be executed using C/C++ operators on the target microcontroller. Even if the nominal MFCC parameters are identical, implementation differences in windowing, fast Fourier transform scaling, Mel filter-bank discretization, log-energy computation, discrete cosine transform, floating-point precision, and feature standardization may lead to numerical differences in the generated feature vectors [13,14,21]. These differences can shift the model input distribution during deployment.

**Figure 2:**
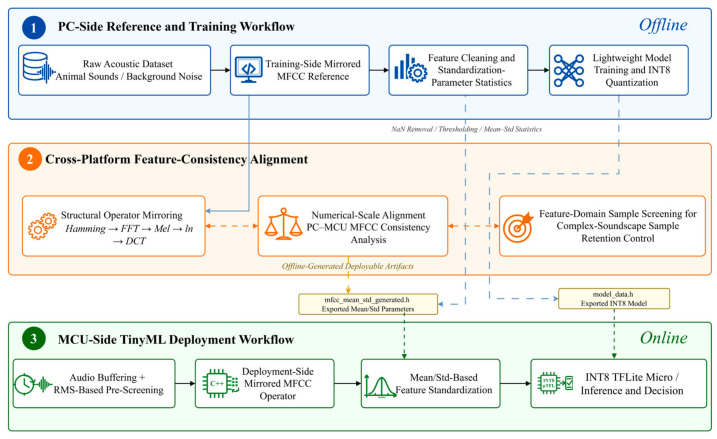
PC-to-microcontroller feature-consistency processing chain for microcontroller-based TinyML acoustic recognition.

To address this issue, this study constructs a PC-to-microcontroller feature-consistency processing chain, as illustrated in Figure 2. The processing chain contains three levels: an offline PC-side reference and training workflow, a cross-platform feature-consistency alignment workflow, and an online microcontroller-side deployment workflow. The offline workflow performs MFCC feature generation, feature-domain filtering, standardization-parameter estimation, lightweight neural-network training, and INT8-quantized model generation. The consistency-alignment workflow constrains the feature-processing procedure through structural operator mirroring, numerical-scale control, and threshold calibration. The online deployment workflow performs audio buffering, amplitude-based triggering, C++ MFCC extraction, exported-parameter-based feature standardization, and TensorFlow Lite Micro inference.

At the structural level, the microcontroller-side MFCC operator is implemented in C++ according to the same parameter configuration used by the training-side reference procedure. The implementation includes Hamming windowing, 256-point fast Fourier transformation, power-spectrum computation, fixed-parameter Mel filter-bank mapping, logarithmic compression, and DCT-II-based cepstral transformation. The PC-side training pipeline uses a corresponding MFCC reference procedure configured with the same sampling rate, frame length, frame hop, number of Mel filters, MFCC dimensionality, and DCT type. This structural alignment reduces uncontrolled differences caused by default settings in high-level acoustic libraries and provides a consistent feature-computation basis for deployment.

At the numerical scale level, this study synchronizes the feature-standardization parameters estimated during training with the microcontroller-side inference pipeline. For each MFCC feature dimension, the training-set mean and standard deviation are estimated on the PC side and exported as C++ arrays. During deployment, the raw MFCC vector generated on the microcontroller is standardized dimension-wise using these exported parameters. The standardization process is expressed as

(3) $$X_{norm,i}=\frac{x_{i}-\mu_{i}}{\sigma_{i}},1\leq i\leq D$$

where $x_{i}$ denotes the i-th raw MFCC feature, $\mu_{i}$ and $\sigma_{i}$ denote the training-set mean and standard deviation of the feature dimension i, respectively, and D denotes the total feature dimensionality. This parameter synchronization helps maintain a consistent input-feature scale between the training and deployment pipelines.

For extremely low-energy or near-silent samples, certain feature dimensions may have small statistical variances. To improve numerical robustness during parameter export, an optional denominator-protection form is reserved:

(4) $$\sigma_{safe,i}=\sigma_{i}+\varepsilon_{s},\;1\leq i\leq D$$

The corresponding safety-aware standardization form is

(5) $$X_{safe,i}=\frac{x_{i}-\mu_{i}}{\sigma_{safe,i}},1\leq i\leq D$$

where $\sigma_{safe,i}$ denotes the safety-adjusted standard deviation and $\varepsilon_{s}$ denotes a configurable positive safety-bias constant. This optional mechanism is used only as a numerical-safety constraint during parameter export and does not change the MFCC feature-extraction pipeline or the neural-network architecture. In the current implementation, the microcontroller performs dimension-wise standardization using the exported mean and standard deviation arrays.

For consistency with the on-device implementation, the vector-form standardization process can be summarized as

(6) $$MFCC_{norm}=\frac{MFCC_{raw}-\mu_{MFCC}}{\sigma_{MFCC}}$$

where $MFCC_{raw}$ denotes the raw MFCC feature vector generated on the microcontroller side, $MFCC_{norm}$ denotes the standardized model input feature vector, $\mu_{MFCC}$ denotes the exported mean array, and $\sigma_{MFCC}$ denotes the exported standard deviation array. The subtraction and division operations are performed element-wise.

Table 2 summarizes the deployment-side standardization-parameter synchronization and optional numerical-safety configuration.

### 3.5. Hardware-Aware Dataset Construction

To reduce the mismatch between the training data and the online deployment environment, this study adopts a hardware-aware dataset construction strategy. In acoustic recognition tasks, recording devices and acquisition front ends may introduce different frequency responses, sampling-path characteristics, and quantization effects. Such acquisition-domain differences can affect the statistical distribution of acoustic features and may degrade model generalization when the training and deployment devices are inconsistent [14]. Therefore, the dataset construction process in this study incorporates the acquisition characteristics of the target deployment hardware, particularly for background-noise and non-target soundscape samples.

The target deployment platform is the Arduino Nano 33 BLE Sense Rev2, and its onboard PDM microphone is used to collect part of the background-noise data. Compared with constructing the background class entirely from external high-performance recording devices, this strategy exposes the model during training to acoustic inputs produced by the same onboard microphone and acquisition path used during deployment. The purpose is not to perform post hoc frequency-response compensation, but to reduce the distributional gap between the training-side background samples and the online microcontroller-side acoustic inputs.

The background-noise class is further expanded using a compound background-noise set to improve coverage of complex non-target soundscapes. Background sounds recorded only with the deployment-side hardware may be insufficient to represent the full range of environmental interference that can occur in field monitoring. Therefore, the dataset incorporates multiple non-target sound types with different physical origins, spectral structures, and temporal dynamics. These include dry-leaf rustling, dry-branch snapping, distant human speech, hydraulic machinery noise, low-frequency thunder, rainfall, and wind noise at different intensity levels. Such environmental-domain expansion is intended to expose the model to broader non-target acoustic distributions rather than merely increasing the number of samples [5,25,26].

For the current experimental configuration, the background-noise category is treated as one compound output class rather than being divided into separate subtypes for classification. The background subtypes are used to improve environmental coverage and to support source-isolated partitioning, but the final recognition task retains a unified background-noise class. This design is consistent with the practical purpose of edge-side monitoring, where non-target acoustic events should be rejected or classified as background rather than identified as fine-grained environmental categories.

The resulting recognition task follows a “6 + 1” seven-class classification scheme. The six target animal classes are white-lipped deer, Tibetan antelope, Tibetan wild ass, wild yak, leopard, and snow leopard. The seventh class is a compound background-noise class representing non-target acoustic events and complex environmental interference. The background-noise samples are organized into three high-level environmental domains: biogenic and biological-material-related sounds, natural geophysical noise, and anthropogenic or mechanical noise. This organization is inspired by the soundscape-ecology concepts of biophony, geophony, and anthrophony [28], while preserving a single background output class for microcontroller-level recognition.

### 3.6. Source-Isolated Dataset Partitioning Protocol

To obtain a more reliable evaluation protocol, this study adopts a source-isolated dataset partitioning strategy. Conventional random shuffling may introduce same-source leakage in acoustic recognition tasks. If derived samples originating from the same recording source, background event, or contiguous slicing sequence are assigned to both the training and test sets, the model may learn source-specific environmental fingerprints rather than transferable acoustic patterns. This can produce overly optimistic test results and weaken the interpretability of the reported generalization performance [23,24].

To reduce this risk, the dataset is partitioned using a source-isolated mode based on filename conventions, source identifiers, session information, and background-subtype labels. For the six target animal classes, samples are assigned to the training or test set according to source identifiers embedded in their filenames. Samples with training-source identifiers are assigned to the training set, whereas samples with test-source identifiers are assigned to the test set. This prevents samples derived from the same source session from appearing in both subsets.

For the background-noise class, the partitioning process combines background-subtype identifiers with filename-based source identifiers. Samples associated with S1 or training-source identifiers are assigned to the training set, whereas samples associated with S2 or test-source identifiers are assigned to the test set. For background subtypes without explicit S1/S2 labels, the assignment is determined according to the training or test source identifiers parsed from the filename. Samples for which no reliable source identifier can be parsed are excluded from the current source-isolated evaluation set.

**Figure 3:**
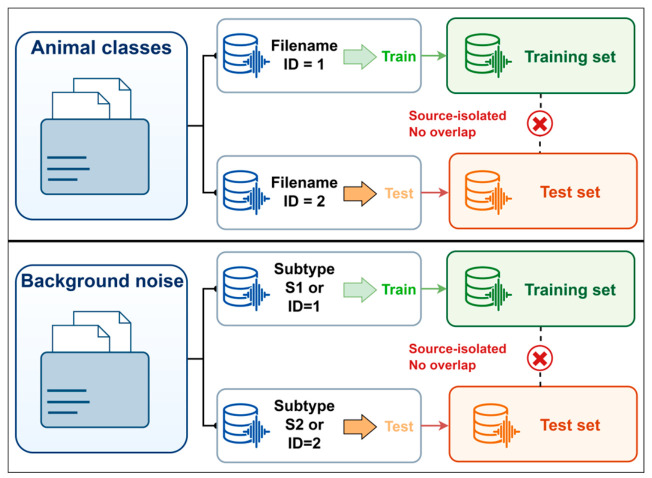
Schematic illustration of the source-isolated partitioning mechanism based on file naming and background-subtype identifiers.

Under this protocol, samples originating from the same source session or the same background event are restricted to one side of the training/test split. This design reduces the risk of cross-set leakage caused by same-source slices and encourages the model to learn acoustic patterns that are more stable across different sources and environmental conditions. The source-isolation mechanism is illustrated in Figure 3.

Table 3 summarizes the final retained training and test sample distribution under the source-isolated evaluation mode.

As shown in Table 3, each target animal class contains 312 valid samples, including 270 training samples and 42 test samples. The compound background-noise class contains 900 valid samples, including 630 training samples and 270 test samples. The complete source-isolated evaluation set therefore contains 2250 training samples and 522 test samples. The enlarged background class is used to improve the coverage of complex non-target acoustic distributions and to support background-discrimination evaluation under realistic soundscape conditions.

### 3.7. Feature-Domain Threshold Calibration and Robustness Analysis Design

To examine the influence of feature-domain numerical filtering on the acoustic recognition pipeline, a Quick Clean threshold-ablation design was used during data preparation. The Quick Clean operator was applied after MFCC extraction and before model training and evaluation. It retained a feature vector only when the vector contained no not-a-number entries and its maximum absolute feature magnitude was lower than a predefined threshold. This operation was designed to control invalid or abnormal feature-domain values rather than to modify raw audio waveforms.

Three feature-domain filtering settings were considered: no amplitude threshold, τ = 128, and τ = 200. The setting without an amplitude threshold was used to characterize model behavior when only numerical validity was checked. The τ = 128 setting represented a more conservative filtering condition, whereas the τ = 200 setting allowed more high-response acoustic feature vectors to be retained. This design was used to evaluate whether the selected threshold excessively removed valid samples or affected performance through feature-domain outlier removal and retained-sample coverage.

The Quick Clean threshold was independent of the deployment-side root-mean-square trigger threshold. The Quick Clean threshold operated on extracted 312-dimensional MFCC feature vectors in the offline data-preparation pipeline, whereas the root-mean-square threshold operated on online audio windows on the microcontroller to determine whether MFCC extraction and neural-network inference should be activated. Therefore, the two thresholds served different purposes: feature-domain numerical validity control and edge-side acoustic-event triggering, respectively.

For the threshold-ablation analysis, the retained training and test sample numbers were recorded under each setting, and the proposed multilayer perceptron was evaluated using test accuracy and macro-F1 score under the same source-isolated protocol. The threshold setting was determined by jointly considering retained-sample coverage, feature-domain abnormal-value suppression, classification performance, and robustness to complex background-noise samples.

### 3.8. TinyML Model Training, Quantization, and On-Device Deployment

The recognition model is trained using the fixed-dimensional MFCC feature vectors generated from the source-isolated dataset. Each input sample is represented by a 312-dimensional feature vector corresponding to 24 analysis frames and 13 MFCC coefficients per frame. Before model training, the feature vectors are standardized using dimension-wise mean and standard deviation parameters estimated from the training data. For the source-isolated evaluation, the scaler is fitted using only the training split, and the test split is transformed using the same parameters. This prevents information from the test set from being used during evaluation.

The neural-network classifier is implemented as a lightweight fully connected model. The input layer receives the 312-dimensional standardized MFCC feature vector. Two hidden dense layers with 64 and 32 units are used, both with rectified linear unit activation. The output layer uses a softmax activation function with seven units, corresponding to the six target animal classes and one compound background-noise class. The model is trained using the Adam optimizer and sparse categorical cross-entropy loss. Early stopping is used to reduce overfitting and to restore the best model weights according to the monitored training loss or validation loss, depending on the training configuration.

For the source-isolated evaluation, the model is trained on the training split and evaluated on the held-out source-isolated test split. The evaluation metrics include overall accuracy, precision, recall, F1-score, and normalized confusion matrix. This evaluation mode is used to report the final recognition performance under the fixed dataset partitioning protocol. After the evaluation configuration is fixed, a separate deployment-oriented model can be generated for microcontroller export using the available training-side feature set and the corresponding standardization parameters. The performance metrics reported in this study are obtained only from the held-out source-isolated test split.

To deploy the trained model on the Arduino Nano 33 BLE Sense Rev2, the trained Keras model is converted into a TensorFlow Lite model and then quantized using post-training INT8 quantization [29]. A representative calibration subset from the training-side standardized features is used during quantization calibration so that the quantizer can observe the numerical range of standardized MFCC inputs. In the current implementation, up to 150 feature vectors are randomly sampled without replacement from the available training-side feature set to form the representative calibration data. The converted model uses 8-bit integer input and output tensors, which reduces model size and improves deployability on the resource-constrained microcontroller.

The final deployment package consists of the quantized model array and the exported standardization parameters. The quantized model is converted into a C/C++ header file and included in the microcontroller firmware. The mean and standard deviation arrays used for MFCC feature standardization are also exported as a C/C++ header file. During on-device inference, the microcontroller acquires an audio window, extracts the raw MFCC feature vector using the C++ mirrored MFCC operator, standardizes the feature vector using the exported parameters, and feeds the standardized input tensor into the TensorFlow Lite Micro interpreter. The inference output is then decoded into the predicted class label and confidence information for edge-side monitoring and response.

## 4. Results

### 4.1. Cross-Platform MFCC Consistency Verification

To verify whether the PC-side training pipeline and the microcontroller-side deployment pipeline can generate consistent acoustic feature representations, a fixed pulse-code modulation (PCM) input sample was used for cross-platform MFCC comparison. The comparison focused on the raw MFCC features generated by the two pipelines under the original setting and the offline energy-scale comparison setting. The purpose of this experiment was to evaluate whether the mirrored MFCC operator and synchronized processing configuration could limit structural and numerical discrepancies between the training and deployment feature pipelines.

**Figure 4:**
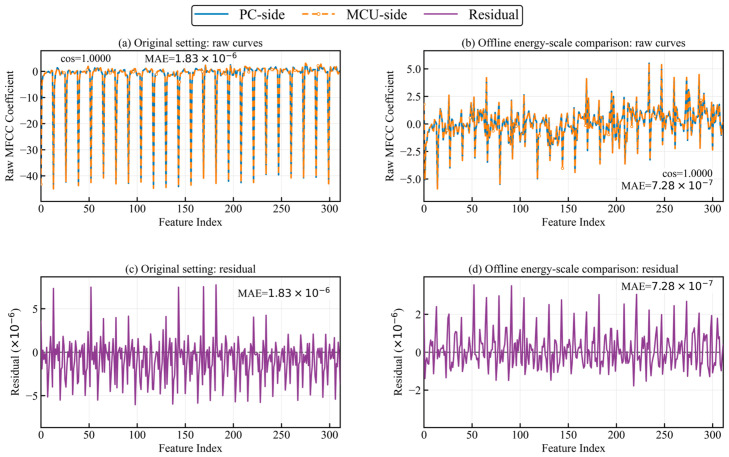
PC-side and microcontroller-side raw MFCC feature curves and residuals under a fixed PCM input: (**a**) raw feature curves under the original setting; (**b**) raw feature curves under the offline energy-scale comparison setting; (**c**) residual curve under the original setting; (**d**) residual curve under the offline energy-scale comparison setting. Residuals are computed as PC-side minus microcontroller-side values and displayed at the $10^{-6}$ scale.

Figure 4 compares the raw MFCC feature curves and residual curves generated by the PC-side and microcontroller-side pipelines. Under the fixed PCM input condition, the two curves show strong overlap under both comparison settings, and the cosine similarities remain close to 1. This result indicates that, after aligning the main MFCC processing steps, including windowing, fast Fourier transformation, Mel filter-bank mapping, log-energy computation, and discrete cosine transformation, the two pipelines can produce highly similar feature patterns. The comparison also shows that power-spectrum scale variation mainly affects the numerical range of low-order MFCC components, particularly those associated with the overall energy level.

**Figure 5:**
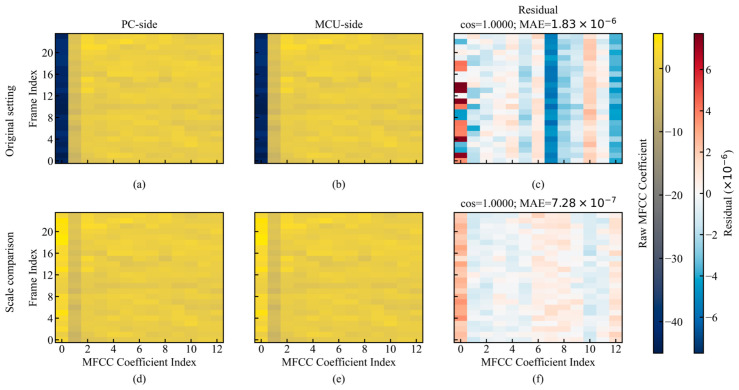
PC-side and microcontroller-side raw MFCC heatmaps and residual heatmaps under a fixed PCM input: (**a**) PC-side MFCC heatmap under the original setting; (**b**) microcontroller-side MFCC heatmap under the original setting; (**c**) residual heatmap under the original setting; (**d**) PC-side MFCC heatmap under the offline energy-scale comparison setting; (**e**) microcontroller-side MFCC heatmap under the offline energy-scale comparison setting; (**f**) residual heatmap under the offline energy-scale comparison setting.

Figure 5 further presents the two-dimensional MFCC heatmaps and residual distributions generated by the two pipelines. The PC-side and microcontroller-side MFCC matrices show highly consistent structural distributions along both the frame dimension and the MFCC-coefficient dimension. Under the original setting, the feature matrices already exhibit similar cepstral patterns, although small local residuals can still be observed. After the offline energy-scale comparison setting is applied, the residual distribution becomes more concentrated around zero, with residual magnitudes on the order of $10^{-6}$. The mean absolute residual decreases from approximately $1.83\times 10^{-6}$ to approximately $0.73\times 10^{-6}$, while the cosine similarities remain close to 1 under both settings.

These results suggest that the proposed PC-to-microcontroller feature-consistency processing chain can generate highly consistent MFCC representations under controlled input conditions. The offline energy-scale comparison further confirms that power-spectrum scaling can influence the numerical range of raw MFCC features. However, this comparison is used only for analyzing cross-platform numerical sensitivity. The final deployment implementation does not introduce an additional online compensation factor in the power-spectrum calculation; instead, the microcontroller-side pipeline continues to use the defined C++ MFCC computation procedure described in Section 3.

### 4.2. Acquisition-Domain Response Difference Analysis

In addition to feature-operator consistency, acquisition-domain differences may also affect the distribution of acoustic features. To examine whether different acoustic front ends introduce observable response differences, a linear-chirp comparison was conducted using a smartphone microphone and the onboard PDM microphone of the Arduino Nano 33 BLE Sense Rev2. This experiment was not intended to provide absolute frequency-response calibration. Instead, it was used to illustrate that different recording front ends can produce different normalized response characteristics under the same excitation and acquisition conditions.

**Figure 6:**
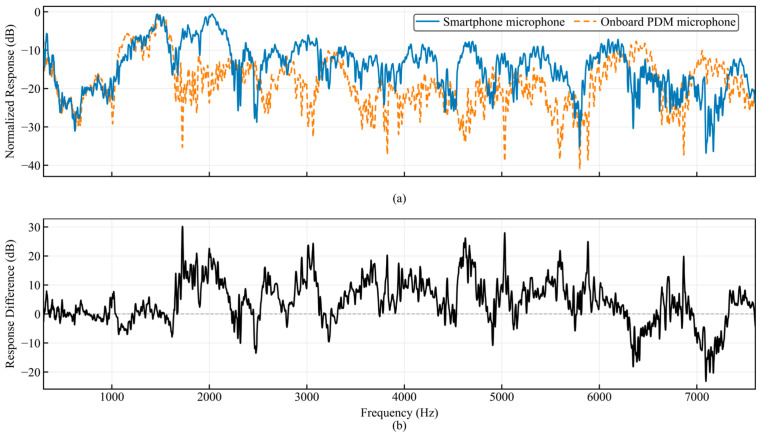
Chirp-based comparison between the smartphone microphone and the onboard PDM microphone: (**a**) normalized relative acoustic responses; (**b**) response difference between the two microphones.

As shown in Figure 6, the normalized relative response curves of the smartphone microphone and the onboard PDM microphone differ across multiple frequency regions. Their response difference also fluctuates over the analyzed frequency range, indicating that the acquisition front end itself can influence the recorded acoustic signal and, consequently, the extracted feature distribution. These observations support the hardware-aware dataset construction strategy used in this study, especially for background-noise and non-target soundscape samples.

These results indicate that using the target deployment hardware to collect part of the background-noise data can help reduce the mismatch between training-side background samples and online microcontroller-side acoustic inputs. Rather than relying on post hoc frequency-response compensation, this study incorporates the acquisition characteristics of the onboard PDM microphone during dataset construction. This design exposes the model to background-class samples that better reflect the response characteristics, sampling path, and quantization effects of the target sensor node, thereby improving the deployment relevance of the training data.

### 4.3. Threshold Calibration Under Complex Soundscapes

To evaluate the influence of complex background interference on the MFCC feature space, a noise-intensity perturbation experiment was conducted using snow leopard samples and strong-wind background noise as an illustrative example. Clean target samples were compared with samples under medium-noise and strong-noise conditions, and the resulting MFCC features were projected using t-distributed stochastic neighbor embedding (t-SNE) [30]. This visualization was used to examine the relative distributional changes between target-animal samples and background-noise samples under increasing interference levels.

**Figure 7:**
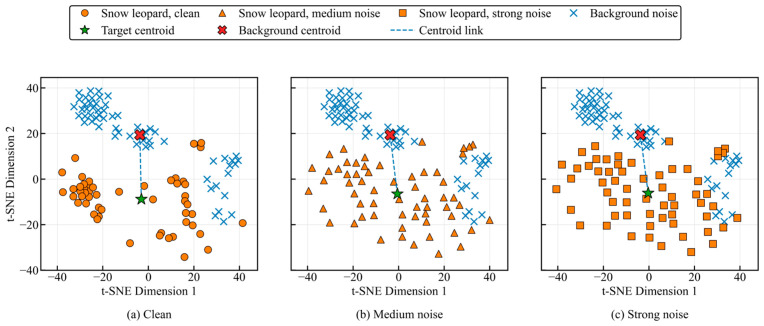
Noise-intensity-dependent changes in the relative distributions of snow leopard and background-noise samples in the t-SNE embedding space: (**a**) clean condition; (**b**) medium-noise condition; (**c**) strong-noise condition. The green star and red cross denote the auxiliary centroids of the target class and background-noise class, respectively, and the blue dashed line indicates their centroid-to-centroid relationship.

As shown in Figure 7, the snow leopard samples remain relatively compact and largely separated from the background-noise cluster under the clean condition. As the noise intensity increases from medium noise to strong noise, the target-class distribution becomes more dispersed, and some snow leopard samples move closer to the background-noise region. This trend suggests that high-energy and non-stationary background components can weaken the separability between target-animal features and background-noise features in the MFCC representation. The observation is consistent with the practical difficulty of animal acoustic recognition in complex field soundscapes, where strong wind, rainfall, and other non-target acoustic events may mask target vocalizations or distort local feature morphology.

On the basis of the noise-perturbation analysis, the feature-domain Quick Clean threshold was further examined using a threshold-ablation experiment. Three settings were compared: no amplitude threshold, τ = 128, and τ = 200. The setting without an amplitude threshold retained samples after checking only numerical validity, whereas τ = 128 and τ = 200 imposed different upper bounds on the maximum absolute MFCC feature magnitude. This comparison was used to determine whether feature-domain filtering improved numerical robustness without excessively removing valid acoustic samples.

**Figure 8:**
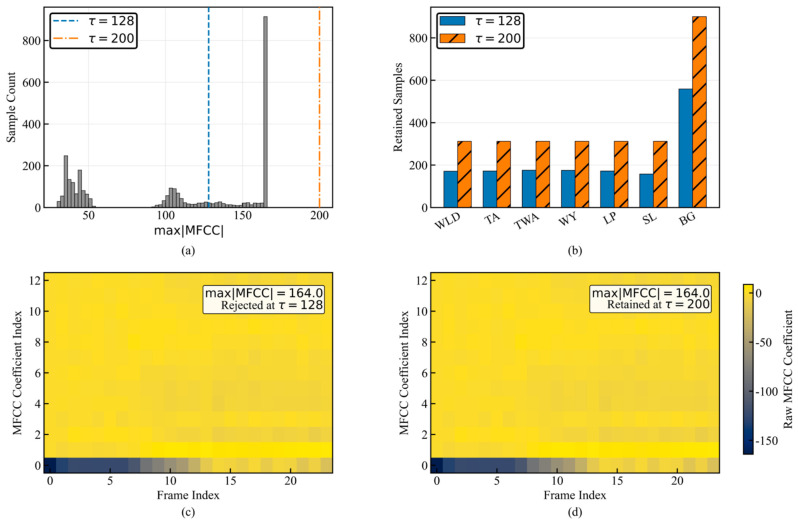
Comparison of feature-domain sample filtering results under different Quick Clean thresholds: (**a**) distribution of sample-wise maximum absolute MFCC values; (**b**) class-wise retained sample counts under τ = 128 and τ = 200; (**c**) MFCC heatmap of the borderline sample rejected at τ = 128; (**d**) MFCC heatmap of the same borderline sample retained at τ = 200. WLD, TA, TWA, WY, LP, SL, and BG denote white-lipped deer, Tibetan antelope, Tibetan wild ass, wild yak, leopard, snow leopard, and background noise, respectively.

Figure 8 compares the sample-wise maximum absolute MFCC values, class-wise retained sample counts, and the retention status of the same borderline sample under the two threshold settings. The distribution of sample-wise maximum feature magnitude contains a high-response interval between τ = 128 and τ = 200, where the retention decision is directly affected by the threshold value. Relaxing the threshold from 128 to 200 increases the number of retained samples across all classes, especially for the compound background-noise class. This result indicates that an overly conservative threshold may remove samples that contain strong background perturbations but still provide useful acoustic information for robust training and evaluation.

The borderline sample shown in Figure 8 further confirms that threshold calibration changes only the sample-screening decision. The sample has a maximum absolute MFCC value of 164.0. Therefore, it is removed under the τ = 128 condition but retained under the τ = 200 condition. The MFCC representation itself remains unchanged. This example illustrates that Quick Clean threshold calibration redefines the valid feature-domain sample range rather than modifying the underlying acoustic signal.

To quantify the effect of different Quick Clean thresholds, Table 4 summarizes the retained sample counts and classification performance under the source-isolated evaluation protocol.

The results show that the setting without an amplitude threshold retained all 522 test samples and achieved a test accuracy of 98.08% and a test macro-F1 score of 97.04%. When τ = 128 was used, the retained test set decreased to 312 samples, indicating that this conservative threshold removed a substantial proportion of valid acoustic samples. Although its test accuracy and macro-F1 score increased slightly, this setting reduced the coverage of complex high-response samples. In contrast, τ = 200 retained all training and test samples in the current source-isolated dataset and achieved the highest test accuracy of 98.85% and test macro-F1 score of 97.95%. Therefore, τ = 200 was selected as the feature-domain Quick Clean threshold because it preserved sample coverage while improving classification performance. The comparison also indicates that the performance improvement was not obtained by removing difficult test samples, since the τ = 200 setting retained the complete test set.

### 4.4. Overall Classification Performance and Confusion Matrix

Using the fixed source-isolated partitioning protocol and the selected Quick Clean threshold of τ = 200, the seven-class acoustic recognition task was evaluated using the proposed multilayer perceptron (MLP) and several baseline classifiers. The task consisted of six target animal classes and one compound background-noise class. Each input sample was represented by a 312-dimensional MFCC feature vector corresponding to 24 frames and 13 coefficients per frame. Following common classification-evaluation practice [31], accuracy and macro-F1 score were used as the main evaluation metrics, because macro-F1 provides a class-balanced measure in the presence of the enlarged background-noise class.

Table 5 compares the proposed multilayer perceptron with representative machine-learning baselines under the same source-isolated evaluation protocol and feature configuration. The decision tree, random forest, support vector machine with a radial-basis-function kernel (SVM-RBF), and k-nearest-neighbor (KNN) classifiers were used as personal-computer-side baselines. In addition, a lightweight convolutional neural network was evaluated using the 24 × 13 MFCC matrix as input to examine whether preserving the two-dimensional MFCC layout improved classification performance.

As shown in Table 5, the random forest baseline achieved the highest test accuracy of 99.04% and the highest test macro-F1 score of 98.29% among the evaluated models. The SVM-RBF and the proposed MLP also achieved strong performance, with test accuracies of 98.66% and 98.28%, respectively. Although the proposed MLP did not achieve the highest personal-computer-side test metric, it was selected as the deployment model because its dense-layer structure can be directly converted into an INT8 TensorFlow Lite Micro model and integrated into the microcontroller-side inference pipeline. In contrast, the random forest, SVM-RBF, and KNN baselines were used for classification comparison on the personal-computer side and were not used as the final microcontroller deployment models in this study.

The lightweight CNN baseline achieved a test accuracy of 92.15% and a test macro-F1 score of 88.45%, which were lower than those of the proposed MLP. This result indicates that, under the current source-isolated dataset and lightweight-model configuration, preserving the two-dimensional MFCC layout using a small CNN did not improve generalization. Therefore, the 312-dimensional flattened MFCC vector combined with the proposed MLP provided a more suitable balance between recognition performance and microcontroller deployability for the current system.

**Figure 9:**
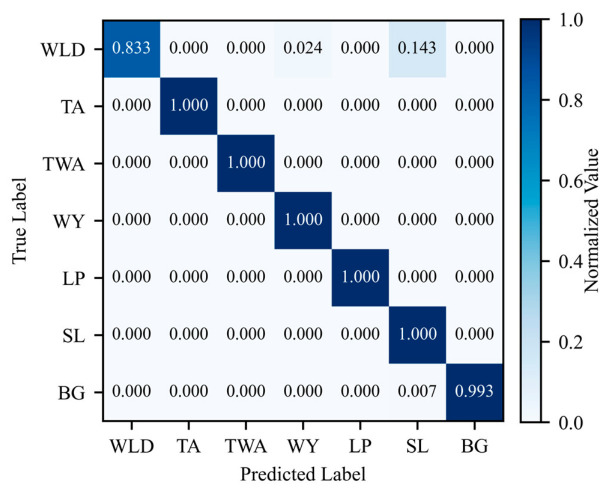
Normalized confusion matrix of a representative MLP run under the source-isolated seven-class evaluation protocol with τ = 200. WLD, TA, TWA, WY, LP, SL, and BG denote white-lipped deer, Tibetan antelope, Tibetan wild ass, wild yak, leopard, snow leopard, and background noise, respectively.

Figure 9 shows the normalized confusion matrix of a representative MLP run under the selected Quick Clean threshold of τ = 200. This run was obtained using a fixed representative seed from the multi-seed stability analysis and is used to illustrate the class-wise confusion structure under the source-isolated evaluation protocol. Most predictions are concentrated along the main diagonal, indicating that the deployment-oriented classifier can distinguish the major animal classes and the compound background-noise class under the current test setting. The remaining errors are mainly associated with the white-lipped deer class and a small number of background-noise samples. Specifically, some white-lipped deer samples were misclassified as snow leopard or wild yak, whereas a small proportion of background-noise samples were assigned to snow leopard. Overall, the confusion pattern indicates that the remaining errors are concentrated in a limited number of class relationships rather than being uniformly distributed across all categories.

Overall, the results show that the proposed MLP provides competitive recognition performance while maintaining a deployment-friendly model structure for TensorFlow Lite Micro. The comparison with traditional machine-learning baselines clarifies that the final model selection was determined not only by personal-computer-side accuracy, but also by compatibility with the microcontroller inference pipeline, quantized deployment, and edge-side resource constraints.

### 4.5. Multi-Seed Stability Analysis of the Proposed MLP

To examine whether the performance of the proposed multilayer perceptron was sensitive to random initialization and data-shuffling variation, a multi-seed stability analysis was conducted under the same source-isolated evaluation protocol and the selected Quick Clean threshold of τ = 200. The model was repeatedly trained using ten random seeds, and the validation and test metrics were summarized using the mean and standard deviation across the repeated runs.

As shown in Table 6, the proposed multilayer perceptron achieved a test accuracy of 98.64% ± 0.26% and a test macro-F1 score of 97.87% ± 0.38% across ten random seeds. The small standard deviations of the test metrics indicate that the model performance was stable under repeated training. The validation metrics also remained high, with a validation accuracy of 99.20% ± 0.53% and a validation macro-F1 score of 99.08% ± 0.64%. These results suggest that the proposed deployment-oriented MLP provides stable recognition performance under the current source-isolated dataset and fixed feature-domain filtering configuration.

### 4.6. Feature Manifold and Acoustic-Boundary Interpretation

To further interpret the class-separation characteristics of the MFCC feature space, t-distributed stochastic neighbor embedding (t-SNE) was applied to the MFCC features, and 95% covariance ellipses were used to visualize local distribution ranges and boundary proximity among classes [30]. This analysis was intended to provide an auxiliary interpretation of the confusion-matrix results rather than a strict statistical decision boundary.

**Figure 10:**
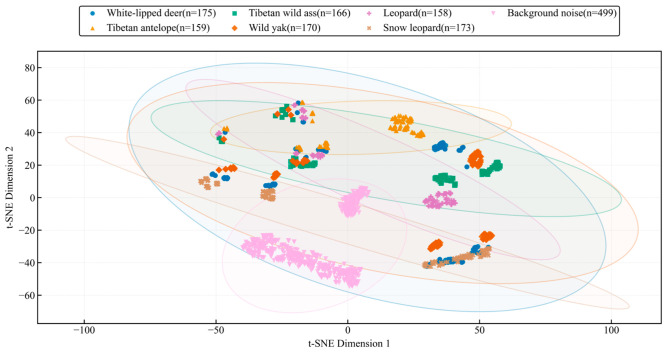
t-SNE visualization of a sampled subset of sample-level MFCC features for the seven-class recognition.

Figure 10 presents the two-dimensional t-SNE visualization of sample-level MFCC features for the seven-class recognition task. Most classes exhibit clustering tendencies to different degrees, indicating that the MFCC representation contains class-discriminative structure in the projected feature space. Tibetan antelope, Tibetan wild ass, and leopard samples show relatively compact local distributions, whereas the background-noise samples form several distinguishable local regions, which is consistent with their largely correct classification behavior in the normalized confusion matrix. The translucent colored ellipses represent the approximate 95% covariance distribution envelopes of different classes in the t-SNE space and are used only as visual aids to indicate class-wise spread and overlap.

The background-noise samples are distributed in relatively distinguishable local regions and show partial separation from several target animal classes. This observation supports the effectiveness of the compound background-noise design for separating non-target acoustic events from the six target animal classes. At the same time, several covariance ellipses overlap to different degrees, indicating that local feature overlap or boundary proximity still exists among several classes.

The local proximity between the white-lipped deer and snow leopard clusters is consistent with the localized confusion pattern observed in Figure 9. This suggests that the remaining misclassifications are not caused by widespread global confusion across all classes, but are mainly concentrated between a few acoustically similar categories. Under the current MFCC representation and lightweight neural-network classifier, such local boundary proximity may be related to short-duration target vocalizations, limited original audio sources, background interference, or similarities in the spectral-envelope structure of some samples.

It should be noted that Figure 10 uses a sampled subset from the current valid experimental dataset to improve visualization clarity. The sample number indicated in the legend represents the number of points used for visualization and is not equivalent to the complete training/test sample size reported in Table 3. Therefore, the t-SNE result should be interpreted as an auxiliary visualization of feature-distribution structure rather than a quantitative substitute for the source-isolated classification metrics.

### 4.7. Edge-Side Resource Utilization and Real-Time Performance

To evaluate the feasibility of the proposed system on a resource-constrained microcontroller platform, edge-side resource utilization and runtime performance were measured on the Arduino Nano 33 BLE Sense Rev2. The evaluation focused on program storage usage, global SRAM usage, model-array size, major runtime workspaces, post-window processing latency, MFCC computation time, TensorFlow Lite Micro invocation time, and trigger behavior under different acoustic conditions. For microcontroller-level TinyML systems, recognition accuracy alone is insufficient; memory footprint, feature-extraction latency, inference latency, and runtime stability must also be considered as system-level deployment indicators.

The deployed classifier was converted into an INT8-quantized TensorFlow Lite Micro model. The model array stored in the generated header file has a length of 27,528 B, corresponding to approximately 26.9 KB. The input feature dimension of the deployed model is 312, corresponding to the 24-frame × 13-coefficient MFCC feature vector generated by the on-device feature-extraction module. This compact model size indicates that the trained classifier can be stored within the flash-memory budget of the target microcontroller.

According to the Arduino compilation output, the complete deployment firmware occupied 374,608 B of program storage, corresponding to 38% of the available flash memory. The global variables occupied 151,312 B of SRAM, corresponding to 57% of the available SRAM. The remaining SRAM reported by the compilation output was 110,832 B, providing a nominal SRAM margin for the stack, local variables, and runtime operations. To measure runtime latency, an instrumented firmware version with serial timing outputs was also compiled. This timing-instrumented firmware occupied 376,112 B of program storage and 151,408 B of global SRAM, indicating that the timing instrumentation introduced only a small additional memory overhead.

**Figure 11:**
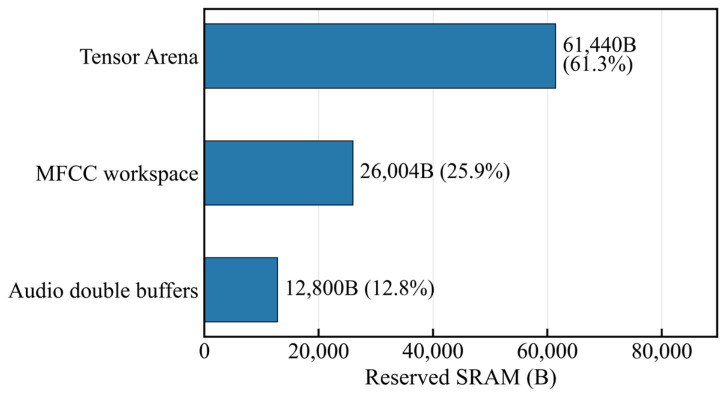
Reserved and estimated SRAM allocation of the major edge-side runtime workspaces. Percentages are computed relative to the sum of the listed workspaces.

Figure 11 illustrates the reserved or statically estimated memory allocation of the three major runtime workspaces: Tensor Arena, MFCC workspace, and audio double buffers. Tensor Arena is the main memory region used by TensorFlow Lite Micro for tensors, intermediate activations, and inference-related runtime buffers. The MFCC workspace supports front-end feature extraction, whereas the audio double buffers support double-buffered audio stream processing. Among the listed workspaces, Tensor Arena accounts for approximately 61.3%, the MFCC workspace accounts for approximately 25.9%, and the audio double buffers account for approximately 12.8%. It should be noted that these percentages are calculated relative to the total size of the three listed workspaces, rather than relative to the entire SRAM capacity or the total global-variable usage reported by the compiler. Therefore, Figure 11 is intended to show the relative distribution among the major runtime components, not to provide a complete dynamic memory profile of the entire firmware.

Table 7 summarizes the edge-side resource utilization of the deployment and timing-instrumented firmware.

Table 8 summarizes the triggering behavior and post-window latency under different acoustic conditions.

As shown in Table 8, the measured post-window processing latency was approximately 303 ms under both animal-sound and background-sound playback conditions. This latency was dominated by MFCC feature extraction, which required approximately 289 ms on average, whereas TensorFlow Lite Micro invocation required only approximately 2.05 ms. Therefore, the main runtime bottleneck of the current implementation is the front-end feature-extraction stage rather than neural-network inference. The result also indicates that further optimization should prioritize the C++ MFCC operator, frame-level spectral computation, and scheduling strategy instead of only reducing the classifier size.

The low-energy monitoring condition produced only one startup transient trigger and did not show stable repeated triggering during the monitoring period. This behavior supports the practical role of the RMS-based pre-inference trigger in suppressing unnecessary model execution during low-energy acoustic periods. However, under acoustic playback conditions, repeated triggering activated the full MFCC extraction and inference pipeline, producing the measured post-window latency reported in Table 8.

Overall, the resource and timing results show that the proposed sensor-based TinyML acoustic monitoring system can be compiled, stored, and executed on the Arduino Nano 33 BLE Sense Rev2. The compact INT8 model, controlled global SRAM usage, and measured runtime behavior support the feasibility of microcontroller-level acoustic recognition under the current experimental configuration. At the same time, the approximately 303 ms post-window latency indicates that feature-extraction optimization remains an important direction for improving responsiveness in future implementations.

### 4.8. RMS Trigger-Threshold Sensitivity Analysis

To examine the effect of the edge-side acoustic trigger threshold on system activation behavior, three RMS trigger thresholds were evaluated: 200, 300, and 400. These thresholds are expressed in raw signed 16-bit PCM amplitude counts after the PDM-to-PCM conversion of the onboard microphone signal; therefore, they represent device-level digital RMS thresholds rather than calibrated sound-pressure-level values. The analysis was conducted under low-energy monitoring, animal-sound playback, and background-sound playback conditions. The first triggered window after device reset was excluded from the stable-stage statistics because it corresponded to a startup transient rather than a stable acoustic input condition. The trigger ratio, accepted ratio, and post-window latency were then calculated from the stable-stage windows.

Table 9 summarizes the trigger-threshold sensitivity results under the three acoustic conditions.

As shown in Table 9, no stable low-energy monitoring window triggered the full recognition pipeline under the three tested thresholds after the startup transient was excluded. The relatively low trigger ratios under animal-sound playback should not be interpreted as low animal-call recall, because the statistics were calculated over all stable 200 ms monitoring windows and the deployed firmware includes a post-inference cooldown interval to avoid repeated activation on adjacent windows from the same acoustic event. Therefore, the trigger ratio mainly characterizes the activation duty of the full MFCC extraction and inference pipeline under continuous monitoring conditions.

Under animal-sound playback, the trigger ratio decreased from 6.25% at an RMS threshold of 200 to 2.08% at an RMS threshold of 400. Under background-sound playback, the trigger ratio decreased from 5.90% to 1.39% as the threshold increased from 200 to 400. Therefore, a lower threshold improved window-level acoustic activation but also increased background-induced triggering, whereas a higher threshold reduced background activation but also suppressed animal-sound activation.

The RMS threshold of 300 provided a practical trade-off between animal-sound activation and background-trigger suppression. Compared with the threshold of 200, it reduced the background-sound trigger ratio from 5.90% to 1.74%, while still preserving a higher animal-sound trigger ratio than the threshold of 400. Therefore, the RMS threshold of 300 was used as the default edge-side trigger threshold in the deployed system. The small differences in post-window latency among the threshold settings mainly reflect runtime variation after a trigger has occurred; the threshold primarily affects whether the full MFCC extraction and inference pipeline is activated.

### 4.9. Power Consumption and Estimated Autonomy

To characterize the energy behavior of the current development-board implementation, system-level input current and power were measured under a 5 V external supply. The measurement was conducted using the normal deployment firmware without serial debug printing, so the measured current better reflected the operating condition of the deployed acoustic monitoring pipeline. Four operating conditions were evaluated: low-energy monitoring, animal-sound playback, background-sound playback, and mixed-operation condition. The estimated autonomy was calculated as

(7) $$T_{autonomy}=\frac{V_{bat}\times C_{bat}\times \eta }{P_{mean}}$$

where $V_{bat}=3.7\;V$, $C_{bat}=2.0\;Ah$, η=0.85, and $P_{mean}$ denotes the measured mean system-level input power.

Table 10 summarizes the measured system-level input power and estimated autonomy under different operating conditions.

As shown in Table 10, the low-energy monitoring condition had a mean input current of 156.56 mA and a mean input power of 0.7832 W. The animal-sound and background-sound playback conditions increased the mean current to 163.57 mA and 164.87 mA, respectively, corresponding to mean input powers of 0.8183 W and 0.8249 W. The mixed-operation condition produced a mean input current of 163.71 mA and a mean input power of 0.8190 W. These results indicate that the additional power increase during acoustically active conditions was moderate compared with the baseline consumption of the development-board implementation.

The estimated autonomy ranged from 7.63 h to 8.03 h under the reference battery setting. The low-energy monitoring condition produced the longest estimated autonomy, whereas background-sound playback produced the shortest estimated autonomy because repeated acoustic activity increased the probability of triggering the full feature-extraction and inference pipeline. These results characterize the power profile of the current development-board implementation rather than an optimized custom low-power sensor node. Further reductions in power consumption would require hardware-level and firmware-level optimization, such as duty cycling, low-power sleep scheduling, peripheral power control, feature-extraction acceleration, or a custom sensing board with a larger energy-storage module.

### 4.10. Unknown Acoustic Input Analysis and Open-Set Boundary

To further examine the behavior of the trained classifier under unseen non-target acoustic inputs, a preliminary confidence-based analysis was conducted using self-recorded unknown sounds that were not included in the training set. Five unknown acoustic types were considered: clap, keyboard typing, knock, phone alert, and whistle. Each sound type contained three raw audio clips, and the clips were segmented into 200 ms windows using the same window length as the main recognition pipeline. The trained model was then used to predict each window, and the predicted class, maximum softmax confidence, background-assignment ratio, target-assignment ratio, and high-confidence target-assignment ratio were recorded.

As shown in Table 11, 60.39% of the unknown acoustic windows were assigned to the compound background-noise class, indicating that this class was able to accommodate a portion of the unseen non-target acoustic inputs. Keyboard typing showed the highest background-assignment ratio of 96.67%, suggesting that this sound type was largely treated as background by the trained classifier. However, other unknown sounds, such as clap and phone alert, produced target-class assignments in more than half of their windows. Overall, 39.61% of the unknown windows were assigned to one of the six target animal classes, and 26.40% produced high-confidence target assignments with a maximum softmax confidence greater than or equal to 0.90.

Because the unknown-input set is limited in size and consists of self-recorded non-target sounds, this analysis should be interpreted as a preliminary diagnostic evaluation rather than as a complete open-set benchmark. These results indicate that the compound background-noise class improves tolerance to some non-target acoustic inputs but does not provide complete open-set or out-of-distribution rejection. Therefore, the current system should be interpreted as a closed-set seven-class classifier with a compound background class, rather than as a complete open-world acoustic event recognition system. For practical field deployment, additional unknown-sound rejection mechanisms, confidence calibration, open-set learning, or hierarchical background modeling may be required to reduce high-confidence target predictions caused by unseen non-target acoustic events.

Table 11 summarizes the confidence-based response analysis under the self-recorded unknown non-target acoustic inputs.

Additional experimental-result tables and edge-side performance logs are provided in the Supplementary Materials.

## 5. Discussion

### 5.1. Practical Implications for Sensor-Based Edge Bioacoustic Monitoring

The results of this study indicate that a complete sensor-based TinyML acoustic recognition pipeline can be implemented on a resource-constrained microcontroller. In contrast to workflows that validate animal sound recognition only on a personal computer, the proposed system establishes an on-device processing loop that integrates acoustic acquisition, MFCC feature extraction, feature standardization, quantized neural-network inference, and edge-side output. Such an implementation is relevant to long-term bioacoustic monitoring, where remote field deployments are often constrained by limited communication bandwidth, limited battery capacity, and high maintenance costs.

The comparative classification results show that the proposed multilayer perceptron achieves competitive recognition performance while remaining compatible with the INT8 TensorFlow Lite Micro deployment pipeline. Although the random forest baseline achieved the highest PC-side test metrics, it was not selected as the final microcontroller deployment model in this study. The proposed MLP was adopted because its dense-layer architecture could be converted into a compact quantized model and integrated into the Arduino Nano 33 BLE Sense Rev2 firmware. This finding indicates that model selection for TinyML acoustic monitoring should not be based solely on classification accuracy, but should also consider model-conversion feasibility, runtime memory usage, inference compatibility, and firmware-level integration.

The multi-seed stability analysis further supports the reliability of the proposed MLP under the current dataset and evaluation protocol. The small standard deviations observed for test accuracy and macro-F1 score suggest that the reported performance was not merely the result of a single favorable random initialization. Together with the source-isolated partitioning protocol and the Quick Clean threshold-ablation analysis, these results provide a more stable assessment of the deployment-oriented classifier.

The cross-platform feature-consistency design also has practical significance. In microcontroller-based acoustic recognition, performance degradation may occur when training-side and deployment-side features are generated by different software libraries or numerical procedures. By aligning MFCC parameters, implementing a mirrored C++ feature-extraction operator, synchronizing standardization parameters, and verifying fixed-input feature consistency, the proposed system reduces an important source of training-to-deployment mismatch. This design improves the engineering reliability of transferring a trained acoustic model from PC-side development to microcontroller-side execution.

### 5.2. Deployment Behavior, Triggering, and Energy Characteristics

The edge-side runtime analysis shows that the current implementation can be compiled, stored, and executed on the Arduino Nano 33 BLE Sense Rev2 while retaining a nominal SRAM margin for runtime operations. The INT8 model size of approximately 26.9 KB, together with the reported flash and SRAM usage, indicates that the complete sensing and inference firmware fits within the resource budget of the target development board. These results support the feasibility of microcontroller-level acoustic recognition under the current feature and model configuration.

The measured post-window latency was approximately 303 ms under acoustic playback conditions. This latency was dominated by MFCC feature extraction, whereas TensorFlow Lite Micro invocation required only a small fraction of the total post-window processing time. Therefore, further improvement in real-time responsiveness should focus primarily on the front-end feature-extraction pipeline, including FFT computation, Mel filter-bank mapping, frame-level scheduling, and memory-access patterns. Reducing only the classifier size would have limited impact on the total post-window latency unless the MFCC computation stage is also optimized.

The RMS trigger-threshold analysis clarifies the role of the edge-side pre-inference trigger. The threshold mainly determines whether the full MFCC extraction and neural-network inference pipeline is activated, rather than changing the processing latency after activation. A lower threshold improves acoustic sensitivity but increases background-induced triggering, whereas a higher threshold suppresses background activation but may also reduce animal-sound activation. Under the tested conditions, the selected threshold of 300 provided a practical trade-off between animal-sound activation and background-trigger suppression.

The power-measurement results should be interpreted as development-board-level system input power rather than chip-level or optimized sensor-node power. The measured input power ranged from approximately 0.783 W to 0.825 W under the tested operating conditions, corresponding to an estimated autonomy of 7.63–8.03 h under the reference battery setting. The relatively small power difference between low-energy monitoring and acoustic playback conditions suggests that the baseline consumption of the development board, continuous microphone acquisition, and monitoring loop dominates the current energy profile. Therefore, future long-duration field deployment will require hardware- and firmware-level energy optimization, such as duty cycling, low-power sleep scheduling, peripheral power control, feature-extraction acceleration, or a custom low-power sensing board.

### 5.3. Background-Noise Discrimination, Limitations, and Generalization Risks

The final source-isolated evaluation shows that the system can distinguish the six target animal classes from the compound background-noise class under the present dataset configuration. This background-discrimination capability is particularly relevant to field monitoring, where non-target acoustic events, such as wind, rainfall, thunder, vegetation friction, distant speech, and mechanical noise, may otherwise cause false alarms or unnecessary data storage. Therefore, the compound background class in this study serves not only as a negative class for model training but also as a practical mechanism for suppressing some non-target acoustic inputs covered by the background class during edge-side monitoring.

However, the unknown acoustic input analysis shows that the compound background-noise class should not be equated with complete open-set or out-of-distribution rejection. Although some unseen non-target sounds were assigned to the background class, a substantial proportion of unknown windows were still assigned to target animal classes, and some produced high-confidence target predictions. This result indicates that the current system should be interpreted as a closed-set seven-class classifier with a compound background class, rather than as a complete open-world acoustic event recognition system. Practical field deployment may therefore require additional unknown-sound rejection mechanisms, confidence calibration, open-set learning, or hierarchical background modeling.

The dataset scale and the number of original acoustic sources also remain limited for some target classes. Data augmentation and compound background expansion can improve sample diversity, but they cannot fully replace the natural distributional variation introduced by different recording distances, seasons, devices, weather conditions, habitats, and individual animals. Therefore, the reported results should be understood as system-level validation under the current dataset and source-isolated protocol, rather than as definitive evidence of generalization across all real field conditions.

Real-time operation on a microcontroller also involves more than classification accuracy. Continuous audio sampling, feature extraction, triggering, and neural-network inference share limited computational resources. If feature extraction occupies the processor for an excessive period, audio buffering may be delayed, increasing the risk of missing short or transient acoustic events. The double-buffer strategy and RMS-based triggering mechanism reduce this risk, but further optimization of scheduling, feature computation, and memory allocation remains necessary for long-duration autonomous deployment.

Overall, this study demonstrates the feasibility of sensor-based TinyML animal acoustic monitoring on a resource-constrained microcontroller, while also showing that practical deployment requires joint consideration of sensing hardware, feature consistency, dataset partitioning, background modeling, runtime latency, power consumption, and unknown-input behavior. The proposed system provides an implementation-oriented foundation for low-maintenance edge bioacoustic monitoring, but broader field validation and more diverse acoustic data are still required before general conclusions can be drawn regarding performance across different deployment scenarios.

## 6. Conclusions

This study presented a sensor-based TinyML acoustic monitoring system for edge-side animal sound recognition on resource-constrained microcontrollers. The system was implemented on the Arduino Nano 33 BLE Sense Rev2 platform and integrated onboard PDM microphone acquisition, MFCC feature extraction, deployment-side standardization, INT8-quantized neural-network inference, and edge-side recognition output. By constructing an end-to-end on-device processing chain, this study demonstrated the feasibility of microcontroller-level acoustic sensing and recognition for low-maintenance animal monitoring scenarios.

To reduce the mismatch between the PC-side training pipeline and the microcontroller-side deployment pipeline, a PC-to-microcontroller feature-consistency processing chain was constructed. The design aligned MFCC extraction parameters, implemented mirrored C++ feature operators, and exported standardization parameters for deployment-side input normalization. Fixed-input consistency verification showed that the two pipelines could generate highly consistent MFCC representations under controlled conditions. In addition, hardware-aware dataset construction, compound background-noise modeling, source-isolated partitioning, and feature-domain Quick Clean threshold calibration were used to improve the deployment relevance and robustness of the evaluation.

Under the fixed source-isolated seven-class evaluation protocol and the selected Quick Clean threshold of τ = 200, the proposed multilayer perceptron achieved 98.28% test accuracy and 97.21% test macro-F1 in the single-run baseline comparison for six target animal classes and one compound background-noise class. The multi-seed stability analysis further showed that the proposed model achieved 98.64% ± 0.26% test accuracy and 97.87% ± 0.38% test macro-F1 across ten random seeds. Baseline comparison indicated that the random forest achieved the highest PC-side test metrics, whereas the proposed MLP was retained as the deployment model because of its compatibility with INT8 TensorFlow Lite Micro conversion and microcontroller-side firmware integration.

The edge-side evaluation showed that the deployed INT8 model occupied 27,528 B, corresponding to approximately 26.9 KB. The normal deployment firmware used 374,608 B of flash storage and 151,312 B of SRAM for global variables, corresponding to 38% and 57% of the available memory resources, respectively. The measured post-window processing latency was approximately 303 ms under acoustic playback conditions, with the latency mainly dominated by MFCC feature extraction rather than neural-network invocation. The system-level input power ranged from 0.783 W to 0.825 W under the tested operating conditions, corresponding to an estimated autonomy of 7.63–8.03 h under the reference battery setting.

The unknown acoustic input analysis showed that the compound background-noise class could accommodate a portion of the unseen non-target acoustic inputs, but it did not provide complete open-set or out-of-distribution rejection. Therefore, the current system should be interpreted as a closed-set seven-class TinyML acoustic classifier with a compound background class, rather than as a complete open-world acoustic event recognition system. Future work will focus on expanding real-world animal sound recordings across different seasons, distances, devices, and environmental conditions; improving cross-source and cross-device generalization; optimizing MFCC computation and power-management strategies; and exploring confidence calibration, open-set rejection, hierarchical background modeling, and multi-sensor cascade triggering for long-duration field deployment.

## Figures and Tables

**Table 1:** MFCC feature-extraction parameters used in this study.

Parameter	Symbol	Value
Sampling rate	$f_{s}$	16 kHz
Analysis window length	$T_{w}$	200 ms
Frame length	$L_{f}$	256 samples
Frame hop	H	128 samples
Overlap ratio	r	50%
Number of frames	$N_{f}$	24
FFT size	$N_{FFT}$	256
Window function	—	Hamming
Number of Mel filters	$N_{m}$	26
MFCC coefficients per frame	$N_{c}$	13
Total feature dimensionality	D	312
Log-stability term	ϵ	$10^{-10}$
Cepstral transform type	—	DCT-II

***Note***: The overlap ratio is calculated as $r=(L_{f}-H)/L_{f}$, and the total feature dimensionality is D=24×13=312. The log-stability term is used in the natural-log energy computation to reduce numerical instability.

**Table 2:** Deployment-side standardization-parameter synchronization and numerical-safety settings.

Parameter	Symbol	Description
Training-set mean	$\mu_{i}$	Training-set mean for dimension-wise standardization
Training-set standard deviation	$\sigma_{i}$	Training-set standard deviation of each feature dimension
Current standardized feature	$X_{norm,i}$	Current deployment-side standardized feature
Safety-bias constant	$\varepsilon_{s}$	Optional positive bias for denominator protection
Safety-adjusted standard deviation	$\sigma_{safe,i}$	Optional safety-adjusted denominator
Safety-aware standardized feature	$X_{safe,i}$	Optional protected form for low-variance feature dimensions
On-device vector form	$MFCC_{norm}$	Vector-form standardization using exported arrays

***Note***: The subscript i denotes the feature-dimension index. The safety-bias constant $\varepsilon_{s}$ is used only as an optional parameter-export constraint for denominator protection. This mechanism does not change the main MFCC feature-extraction pipeline or the model architecture.

**Table 3:** Training and test sample distribution under the source-isolated evaluation mode.

Class	Training	Test	Total
White-lipped deer	270	42	312
Tibetan antelope	270	42	312
Tibetan wild ass	270	42	312
Wild yak	270	42	312
Leopard	270	42	312
Snow leopard	270	42	312
Background noise	630	270	900
Total	2250	522	2772

***Note***: The background-noise samples include biogenic and biological-material-related sounds, natural geophysical noise, and anthropogenic or mechanical noise.

**Table 4:** Quick Clean threshold-ablation results under the source-isolated evaluation protocol.

Quick Clean Setting	Retained Training Samples	Retained Test Samples	Test Accuracy (%)	Test Macro-F1 (%)
No amplitude threshold	2250/2250	522/522	98.08	97.04
τ = 128	1347/2250	312/522	98.40	97.32
τ = 200	2250/2250	522/522	98.85	97.95

**Table 5:** Single-run classification performance comparison under the source-isolated evaluation protocol.

Model	Feature Representation	Validation Accuracy (%)	Validation Macro-F1 (%)	Test Accuracy (%)	Test Macro-F1 (%)	Deployment Role
Decision Tree	312-D MFCC vector	94.89	94.32	97.51	96.47	PC-side baseline
Random Forest	312-D MFCC vector	99.11	98.94	99.04	98.29	PC-side baseline
SVM-RBF	312-D MFCC vector	98.89	98.68	98.66	97.60	PC-side baseline
KNN (k = 5)	312-D MFCC vector	97.56	97.60	96.74	96.32	PC-side baseline
Proposed MLP	312-D MFCC vector	98.89	98.68	98.28	97.21	MCU-deployable INT8 model
Lightweight CNN	24 × 13 MFCC matrix	95.78	95.16	92.15	88.45	PC-side matrix-input baseline

**Table 6:** Ten-seed stability of the proposed multilayer perceptron under the source-isolated evaluation protocol.

Metric	Mean ± Standard Deviation
Validation accuracy (%)	99.20 ± 0.53
Validation macro-F1 (%)	99.08 ± 0.64
Test accuracy (%)	98.64 ± 0.26
Test macro-F1 (%)	97.87 ± 0.38

**Table 7:** Edge-side resource utilization of the deployment and timing-instrumented firmware.

Evaluation Item	Value	Note
INT8 model array size	27,528 B (approximately 26.9 KB)	Stored as a C/C++ model header
Deployment firmware flash usage	374,608 B (38%)	Normal deployment firmware
Deployment firmware global SRAM usage	151,312 B (57%)	Normal deployment firmware
Remaining SRAM reported by compilation	110,832 B	Normal deployment firmware
Timing-instrumented firmware flash usage	376,112 B (38%)	Firmware with serial timing outputs
Timing-instrumented firmware global SRAM usage	151,408 B (57%)	Firmware with serial timing outputs
Instrumentation overhead	+1504 B flash; +96 B SRAM	Relative to the deployment firmware
Input feature dimension	312	24 frames × 13 MFCC coefficients

***Note***: The global SRAM usage refers to the compiler-reported global-variable memory occupation. It is different from the relative workspace distribution shown in Figure 11, which only describes the listed major runtime workspaces.

**Table 8:** Edge-side triggering and post-window latency under different acoustic conditions.

Acoustic Condition	Number of Windows	Trigger Ratio (%)	Accepted Ratio (%)	Avg. Post-Window Latency (ms)	P95 Latency (ms)	Max Latency (ms)	Avg. MFCC Time (ms)	Avg. TFLite Micro Invoke Time (ms)
Low-energy monitoring	950	0.105	0.105	—	—	—	—	—
Animal-sound playback	500	6.400	6.400	302.557	302.678	303.147	288.931	2.048
Background-sound playback	550	7.091	5.636	302.572	302.717	302.975	288.905	2.070

***Note***: The latency values denote post-window processing time and do not include the 200 ms audio-window formation time. P95 denotes the 95th-percentile latency. The low-energy monitoring condition contained only a startup transient trigger; therefore, stable low-energy windows were not used for latency averaging.

**Table 9:** Sensitivity of the RMS trigger threshold under different acoustic conditions.

RMS Threshold	Acoustic Condition	Stable Windows	Triggered Windows	Accepted Windows	Trigger Ratio (%)	Accepted Ratio (%)	Avg. Post-Window Latency (ms)
200	Low-energy monitoring	288	0	0	0.00	0.00	—
200	Animal-sound playback	288	18	18	6.25	6.25	308.09
200	Background-sound playback	288	17	17	5.90	5.90	308.22
300	Low-energy monitoring	288	0	0	0.00	0.00	—
300	Animal-sound playback	288	15	13	5.21	4.51	302.52
300	Background-sound playback	288	5	5	1.74	1.74	302.66
400	Low-energy monitoring	288	0	0	0.00	0.00	—
400	Animal-sound playback	288	6	6	2.08	2.08	308.35
400	Background-sound playback	288	4	4	1.39	1.39	308.31

***Note:*** The RMS threshold is expressed in raw signed 16-bit PCM amplitude counts after PDM-to-PCM conversion. The first triggered window after device reset was excluded from the stable-stage statistics because it corresponded to a startup transient. Accepted windows denote triggered windows that passed the event-acceptance condition and were used for subsequent post-window processing statistics. The trigger ratio denotes the proportion of all stable 200 ms monitoring windows that activated the full MFCC extraction and inference pipeline under the implemented RMS trigger and post-inference cooldown logic; therefore, it should be interpreted as a window-level activation-duty metric rather than as an event-level animal-call recall metric. The latency values denote post-window processing time and do not include the 200 ms audio-window formation time.

**Table 10:** System-level power consumption and estimated autonomy under different operating conditions.

Operating Condition	Duration (s)	Mean Voltage (V)	Mean Current (mA)	Mean Power (W)	Estimated Autonomy (h)
Low-energy monitoring	180	5.0026	156.56	0.7832	8.03
Animal-sound playback	180	5.0030	163.57	0.8183	7.69
Background-sound playback	180	5.0030	164.87	0.8249	7.63
Mixed-operation condition	300	5.0026	163.71	0.8190	7.68

***Note:*** The measured values represent the system-level input power of the Arduino Nano 33 BLE Sense Rev2 development-board implementation under a 5 V external supply. The mean power was calculated as $P_{mean}=V_{mean}\times I_{mean}$, where the current was converted from mA to A. The autonomy was estimated as $T_{autonomy}=\frac{V_{bat}\times C_{bat}\times \eta }{P_{mean}}$, using a 3.7 V, 2.0 Ah reference battery and a conversion efficiency of 0.85; therefore, it should be interpreted as an engineering estimate rather than a full-discharge field-test result.

**Table 11:** Confidence-based response analysis under unknown non-target acoustic inputs.

Unknown Sound Type	Number of Clips	Number of Windows	Mean Maximum Confidence	Background Assignment	Target Assignment	High-Confidence Target Assignment
Clap	3	91	0.878	32/91 (35.16%)	59/91 (64.84%)	38/91 (41.76%)
Keyboard typing	3	90	0.990	87/90 (96.67%)	3/90 (3.33%)	2/90 (2.22%)
Knock	3	43	0.900	25/43 (58.14%)	18/43 (41.86%)	13/43 (30.23%)
Phone alert	3	91	0.923	41/91 (45.05%)	50/91 (54.95%)	34/91 (37.36%)
Whistle	3	41	0.953	30/41 (73.17%)	11/41 (26.83%)	7/41 (17.07%)
Overall	15	356	0.929	215/356 (60.39%)	141/356 (39.61%)	94/356 (26.40%)

***Note:*** High-confidence target assignment denotes windows assigned to one of the six target animal classes with a maximum softmax confidence not lower than 0.90. The maximum softmax confidence is used here as a diagnostic response score rather than as a calibrated probability. These unknown sounds were not used during model training and were introduced only for preliminary response analysis under unseen non-target acoustic inputs.

## Data Availability

The data presented in this study are available from the corresponding author upon reasonable request.

## Supplementary Materials

The following supporting information can be downloaded at: https://www.mdpi.com/article/10.3390/s26133972/s1. Supplementary File S1: Additional experimental-result tables and edge-side performance logs.

## Abbreviations

The following abbreviations are used in this manuscript:

AI	Artificial intelligence
DCT	Discrete cosine transform
DCT-II	Type-II discrete cosine transform
FFT	Fast Fourier transform
INT8	8-bit integer
MCU	Microcontroller unit
MEMS	Microelectromechanical systems
MFCC	Mel-frequency cepstral coefficients
PC	Personal computer
PCM	Pulse-code modulation
PDM	Pulse-density modulation
RMS	Root-mean-square
SRAM	Static random-access memory
TFLite	TensorFlow Lite
TinyML	Tiny machine learning
t-SNE	t-distributed stochastic neighbor embedding
